# 

**Published:** 1871-04

**Authors:** 


### INDEX OF SUBJECTS.

### ORIGINAL COMMUNICATIONS.

Hypodermic Administration of Medicinal Agents........ ....................159

### EXTRACTS AND GLEANINGS.

Skin-Grafting, 168; Bromide of Potassium in Ague—Sulphurous Acid in Typhoid Fever, 169; Calabar Bean in Constipation—Etiology and Treatment of Diptheria, 170; Arsenic—Sloughing Syphilitic Sore Throat—Chronic Catarrh, 171; Treatment of Gonorrhœa by local means, 172; Treatment of Diarrhœa—Hydrangea Aborescens, 173; Creosote—Malarial Fever—Iron and Quinia applied locally in Erysipelas—Phlegmasia Dolens treated by opium, 174; On the treatment of Sciatica—Chloral to Children—Anæsthetic property of Carbolic Acid, 175; Carbolic Acid in Carbuncle—Digitalis in Orchitis, 176; Management of Pertussis—Bromide of Potassium in Dentition, 177; a favorite Tonic Remedy—Treatment of Diarrhœa in Infants—Carb. Ammonia in Pneumonitis, 178; Hydrate of Chloral—Gelseminum semper-virens, 179; Treatment of Diptheria—Perchloride of Iron in Urinary Diseases—A good Anæsthetic Mixture, 180; Embrocation for Inflammation of Breasts—Sulphite of Soda in Chronic Systitis—Bromide of Potassium in Leucorrhœa—Suppression of Urine, 181; Prescriptions for Dropsy, Colliquative Sweats, Prurigo, 182; Syphilitic White Swelling—Carbonate of Iron in Menorrhagia, 183; Prescriptions for Acute Rheumatism, Chronic Chills, Hemorrhoids, Tonsillitis, 184; Colic in Infants—Indolent Ulcers and Suppurative Surfaces—Excellent Effervescing Iron Powder—New Treatment of Sprains, 185.

### MISCELLANEOUS CORRESPONDENCE.

Letter from Prof. J. G. Thomas, of Savannah, Ga...........................186
Our Carolina Correspondent.......................................187
Kentucky Correspondent...........................................190
Savannah Correspondent...........................................191

### EDITORIALS, REVIEWS, ETC.

Professional Brotherhood.........................................193
Shall Medical Societies enforce their Laws, etc..................199
National University..............................................203
The American Medical Association.................................208
Professional Remuneration........................................209
Advertising Patrons—The Macon Medical Association—The Fulton County Medical Society..........................................................212
Our Exchanges....................................................213
Bibliographical..................................................215

